# A dosimetric evaluation of intensity modulated radiotherapy and three-dimensional conformal radiotherapy for prostate cancer in Ghana

**DOI:** 10.3332/ecancer.2024.1707

**Published:** 2024-05-31

**Authors:** Kofi Adesi Kyei, Joseph Daniels, Ameyaw Kwame Adom, Philip Odonkor, Andrew Yaw Nyantakyi, Dorothy Ekua Adjabu

**Affiliations:** 1 Department of Radiography, University of Ghana, Legon, KB 143, Ghana; 2 National Centre for Radiotherapy, Oncology and Nuclear Medicine, Korle-Bu Teaching Hospital, Accra, KB 369, Ghana; 3 Department of Physiotherapy, School of Biomedical and Allied Health, University of Ghana, Accra, KB 143, Ghana; a https://orcid.org/0000-0003-3485-5368; b https://orcid.org/0000-0002-1466-150X; c https://orcid.org/0009-0004-1999-3767; d https://orcid.org/0009-0003-8046-9078; e https://orcid.org/0000-0003-0742-6007

**Keywords:** radiotherapy, 3-D conformal radiotherapy, intensity-modulated radiotherapy, conformity index, homogeneity index, dosimetric evaluation

## Abstract

External beam radiotherapy incorporates treatment techniques such as three-dimensional conformal radiotherapy (3DCRT), intensity-modulated radiotherapy (IMRT), image-guided radiotherapy and volumetric modulated arc therapy to deliver high-energy radiation to cancer. The use of IMRT for cancer treatment is also associated with significant costs for patients in low–middle-income countries. The purpose of this study was to compare the dosimetric properties of 3DCRT and IMRT treatment plans for the external beam irradiation of patients with prostate cancer (Pca) to ascertain the superiority of IMRT in terms of dose homogeneity, conformity and dose limitation to organs at risk (OAR) in a resource-limited setting. One hundred and sixty treatment plans for 80 patients were created using 3DCRT and IMRT on the Eclipse treatment planning system (version 13.6). Data were collected and assessed from the dose-volume histogram of each plan. The conformity and homogeneity index (HI) for each of the plans were calculated. The doses to the OAR were also recorded and evaluated. The mean HIs for the IMRT and 3DCRT treatment techniques were 0.04 ± 0.02 (range: 0.01–0.011) and 0.09 ± 0.02 (range: 0.04–0.016), respectively. The mean conformity index (CI) for IMRT and 3DCRT techniques were 1.257 ± 0.112 (range: 0.99–1.58) and 1.302 ± 0.196 (range: 1.10–2.26). IMRT had a better significant mean HI and CI compared to 3DCRT. Generally, for this study, IMRT had better organ sparing compared to 3DCRT. The mean doses for the OARs ranged from 4.3–74.6 Gy for IMRT and 3.1–75.9 Gy for the 3DCRT technique. Overall, this study demonstrates that IMRT may offer an enhanced therapeutic profile, potentially reducing toxicity to the patient and ensuring more precise dose delivery to the target volume compared to 3DCRT in PCa external beam irradiation.

## Introduction

### Background

Prostate cancer (PCa) is the fourth most frequently diagnosed malignancy worldwide after breast, lung and colorectal cancers. PCa is also the seventh leading cause of cancer-related deaths with 375,304 mortalities reported globally in 2020 alone [1]. In all, 26,392 new cases were diagnosed in West Africa with an incidence of ≥200 cases per 100,000 population per year reported in Ghana [2]. Contemporary modalities for the management of PCa include surgery, radiotherapy (RT), chemotherapy, cryotherapy, hormonal therapy and immunotherapy [3,4]. Compared with other treatment modalities, RT offers more desirable local tumour control, disease-free survival and side-effect profile [5]. RT may be delivered as definitive therapy in the form of teletherapy or brachytherapy and can be used in conjunction with other treatment modalities depending on the stage and risk stratification of the disease [6].

External beam radiotherapy (EBRT) incorporates treatment techniques such as three-dimensional conformal radiotherapy (3DCRT), intensity-modulated radiotherapy (IMRT), image-guided radiotherapy and volumetric modulated arc therapy [7]. The use of different treatment planning (TP) techniques helps to produce better dose distribution within patients thereby minimising the adverse effects of radiation in normal tissues [8, 9]. Advances in computer hardware and software technology have enabled the implementation of improved algorithms for dose calculation and optimisation, as well as the development of several complex treatment approaches [10]. TP progressed from 2-dimensional planning based on pelvic bone landmarks on X-ray fields to 3DCRT planning based on computed tomography (CT) [11]. 3DCRT conforms doses to the target of irradiation and is adopted as the gold standard in cancer treatment due to its superior target coverage and lower toxicity to normal organs at risk (OAR) than two-dimensional radiotherapy.

The basic premise of IMRT is multi-directional irradiation with non-uniform energy fluence beams designed to give a high dose to the target volume while delivering an acceptable dosage to the surrounding normal structures [12], which is achieved by breaking each treatment beam into smaller beam segments with the aid of multi-leaf collimators (MLCs) [13, 14]. IMRT is more effective than 3DCRT in terms of target coverage, dose uniformity and the sparing of OARs from RT-induced damage [15].

In many resource-limited settings, EBRT for the treatment of PCa is achieved primarily with 3DCRT due to the unavailability of IMRT. The use of IMRT for cancer treatment is also associated with significant costs for patients in low–middle-income countries. This comparative study was conducted at a major cancer treatment center in sub–Saharan Africa to evaluate the dosimetric properties of 3DCRT and IMRT treatment plans for the external beam irradiation of patients with PCa to ascertain the superiority of IMRT in terms of dose homogeneity, conformity and dose limitation to OARs in a resource-limited setting.

## Methods

This research was a cross-sectional study conducted at a major radiation treatment centre in the capital city of a low–middle-income country in sub-Saharan Africa where both 3DCRT and IMRT techniques are routinely used for the treatment of patients with PCa. Simple random sampling was used to select 80 eligible patients with localised disease between August 2021 and January 2022. Patients with lymph node-positive disease or distant metastasis were excluded from the study. Each patient underwent CT simulation in the supine position with a pillow and knee rest for comfort and pelvic stability. The CT simulator used was GE Discovery CT590 RT (16 slice count; software version 11BW).

Patients’ clinical and treatment-related data were extracted from an Eclipse treatment planning system (version 13.6). The dose parameters from the dose volume histogram (DVH) were also recorded. An Excel data sheet was used to record the following parameters: D2 [the dose delivered to 2% volume of the planning target volume (PTV)], D50 (the dose delivered to 50% volume of the PTV), D98 (the dose delivered to 98% volume of the PTV), D95 (the dose delivered to 95% volume of the PTV) and V95 (volume of the PTV receiving 95% of the prescribed dose). D2 and D98 represent the near maximum and near minimum doses for the PTV, respectively, as described in the 83rd report of the International Commission on Radiation Units and Measurements (ICRU 83). The mean and maximum doses to the OARs (rectum, bladder and femoral heads) were also recorded. The homogeneity index (HI) and conformity index (CI) of each treatment plan were calculated using the following formula:

$$\mathrm{HI}\;=\;\frac{D2\%-D98\%}{D50\%}$$

$$\mathrm{CI}\;=\;\frac{V95}{\mathrm{VPTV}}$$

where

D2% = dose delivered to 2% of the PTV (near maximum),

D98% = dose delivered to 98% of the PTV (near maximum),

D50% = dose delivered to 50% of the PTV,

V_95_ = volume of the 95% isodose,

VPTV = Volume of the PTV.

**Figure 1.**
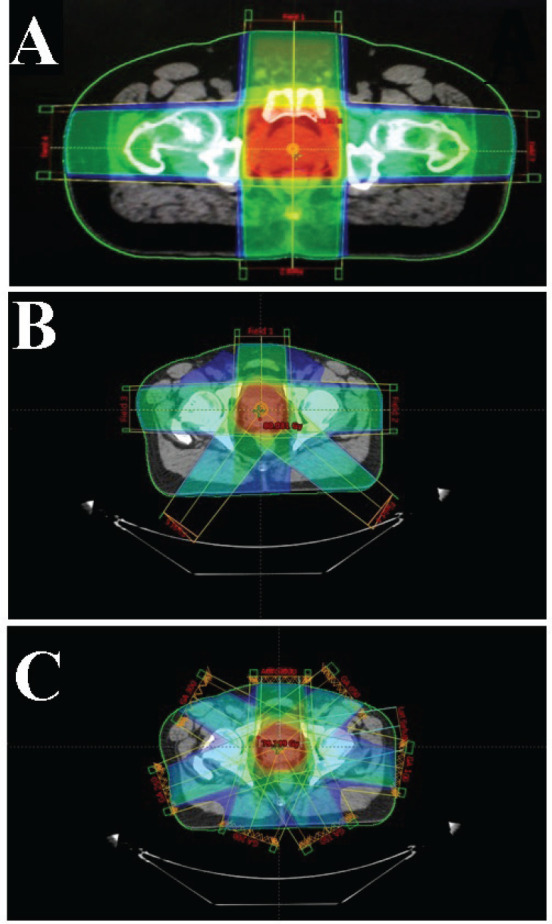
Beam geometries of the four-, five- and seven-field plans generated for the treatment with 3DCRT and IMRT. (a): *A four-field beam arrangement set-up for the 3DCRT plan*. (b): *A five-field beam arrangement set-up for the 3DCRT plan*. (c): A seven-field beam arrangement set-up for the IMRT plan.

New 3DCRT and IMRT plans were created for each study participant while respecting the appropriate dose constraints for all OARs. Doses of 78, 76 and 74 Gy in daily fractions of 2 Gy to be delivered from Monday to Friday over 8 weeks were prescribed for the two sets of plans. A seven-field beam plan with gantry angles of 0^0^, 50^0^, 100^0^, 150^0^, 200^0^, 250^0^ and 300^0^ was prepared using 6 MV Linac photons for IMRT. Similarly, four-field (0^0^, 90^0^, 180^0^ and 270^0^) and five-field (0^0^, 90^0^, 142^0^, 215^0^ and 270^0^) beam plans were also generated for the 3DCRT plans as summarised in Appendix I. The varying beam arrangement for the 3DCRT plans was used to achieve the best possible plan (best coverage to the PTV and lower dose to the OARs, meeting all tolerances) that met all requirements for the treatment of patients. Figure 1 demonstrates the beam geometry of the four-, five- and seven-field plans which were generated for the two sets of treatment plans. The dose constraints adopted for the various OARs are also summarised in Appendix II.

Data were analysed with the Statistical Package for the Social Sciences software version 20.0. Inferential and descriptive analyses were performed including frequencies, SD, means and interquartile range. The results were presented using tables and clustered charts. A paired sample *t*-test at a significance level of 0.05 was performed to compare the means of parameters of the two sets of radiation treatment plans. All patients’ data were anonymised and handled with utmost confidentiality. Informed consent was acquired from the patients whose data were selected for this study. Permission was obtained from the management of the healthcare facility where this study was conducted. Ethical approval was obtained from the institutional review board before the commencement of the study. The study was conducted after institutional review board approval. Written informed consent was obtained from all patients before the use of their medical information in this study. The confidentiality and privacy of the participants were always maintained during this study. Patients’ identifying information was removed from all extracted medical records. The study adhered to all stipulated ethical principles and standards, including the Declaration of Helsinki.

## Results

### Prescribed dose and treatment-field parameters

**Figure 2.**
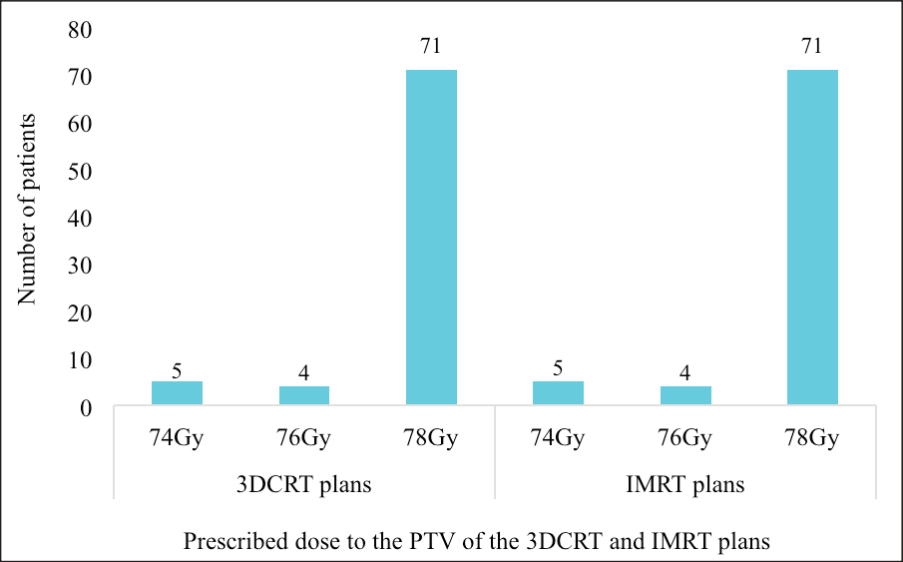
The various prescribed doses to the PTV of both sets of treatment plans. 3DCRT = 3-dimensional conformal radiotherapy, IMRT = intensity-modulated radiotherapy, PTV = planning target volume.

The study involved radiation treatment plans of 80 unique patients. One 3DCRT and one IMRT plan were generated for each patient. Hence a total of 160 plans (80 3DCRT and 80 IMRT) were analysed in total. Figure 2 illustrates the dose and field parameters of the various treatment plans. In all, 71 (88.8%), 4 (5.0%) and 5 (6.2%) of both the 3DCRT and IMRT plans were prescribed to PTV doses of 78, 76 and 74 Gy, respectively.

**Figure 3.**
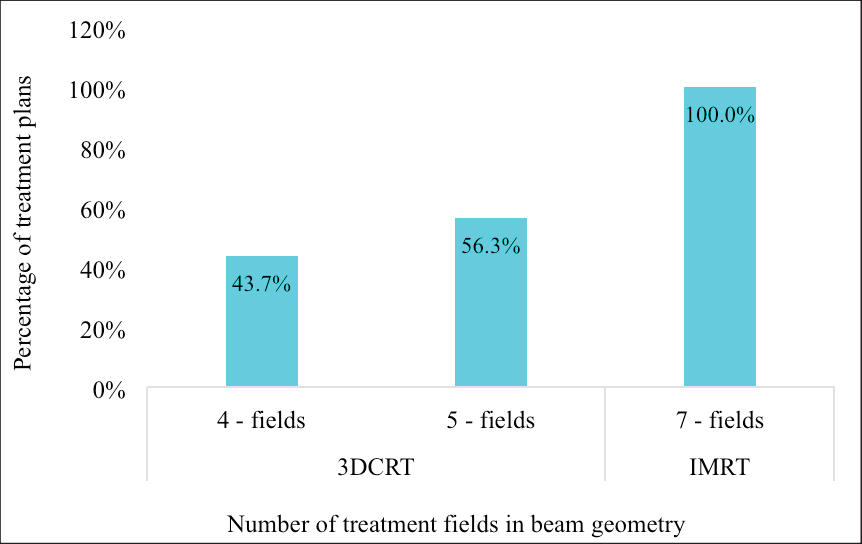
The proportion of four-, five- and seven- field beam geometry generated for both the 3DCRT and IMRT treatment plans. 3DCRT = 3-dimensional conformal radiotherapy, IMRT = intensity modulated radiotherapy.

All the IMRT plans generated involved seven fields whereas the 3DCRT plans involved either four (*n* = 35, 43.8%) or five fields (*n* = 45, 56.3%) which were used to be able to meet the dose constraints of the OARs (Figure 3).

### Dose parameters of the PTV

#### Target coverage

The target coverage was defined as D95 (dose delivered to 95% of the target volume). The mean coverage of D95 for IMRT (98.6% ± 1.1%) was higher than that for 3DCRT (96.1% ± 0.90%).

#### HI (doses to 2%, 50% and 98% of the target volume)

The mean dose for D2 was 79.16 ± 1.72 Gy for IMRT and 80.20 ± 1.35 Gy for 3DCRT. The D50 value for IMRT was 77.86 ± 1.47 Gy whereas that for 3DCRT was 77.93 ± 1.00 Gy. A higher D98 mean dose of 75.97 ± 1.66 Gy for IMRT was recorded compared to 73.11 ± 1.51 Gy for 3DCRT (Table 1). The mean HI for the IMRT and 3DCRT techniques were 0.04 ± 0.02 and 0.09 ± 0.02, respectively. The range was 0.01–0.011 and 0.04–0.016 for IMRT and 3DCRT, respectively. The mean HI for IMRT was more homogeneous than for 3DCRT.

#### CI (Target volume versus 95% isodose volume)

The mean target volume for this study was 222.30 ± 82.19 cc. The mean volumes receiving 95% of the prescribed dose (as indicated by the 95% isodose lines) were 275.96 ± 95.36 cc and 284.66 ± 99.71 cc for IMRT and 3DCRT, respectively, as shown in Table 1. The mean CI was calculated to be 1.257 ± 0.112 and 1.302 ± 0.196 for IMRT and 3DCRT, respectively.

#### Dose parameters of the critical OAR

The OARs were evaluated per the constraints provided in Appendix II. The mean doses of the OARs are presented in Table 2.

### Rectum

Overall, 50% volume of the rectum received mean doses of 45.78 ± 5.26 Gy and 51.12 ± 5.35 Gy with the use of IMRT and 3DCRT, respectively. Also, 30% volume of the rectum received a mean dose of 59.51 ± 6.16 Gy with IMRT and 59.72 ± 6.95 Gy with 3DCRT.

### Bladder

For IMRT and 3DCRT, 50% of the bladder volume received mean doses of 38.12 ±13.35 and 38.18 ± 18.77 Gy, respectively. Also, 30% of the volume received mean doses of 55.87 ±13.14 and 51.73 ±16.28 Gy with IMRT and 3DCRT, respectively.

### Femoral heads

Overall, 5% volume of the right femoral head received mean doses of 34.61 ± 6.41 and 39.71 ± 5.34 Gy with IMRT and 3DCRT, respectively. Also, 5% volume of the left femoral head received mean doses of 34.61 ± 6.41 and 39.71 ± 5.34 Gy with IMRT and 3DCRT, respectively.

## Discussion

### Homogeneity index

This study compared the HI for the two treatment techniques using the ICRU 83 formulae. HI closer to zero is indicative of a better homogeneity. In this study, the measured mean HI for IMRT was 0.04 ± 0.02 whereas that for 3DCRT was 0.09 ± 0.02. IMRT was associated with better homogeneity that was statistically significant (*p* = 0.000). This is consistent with the findings of Salimi *et al* [7]. The H_1_ of Adeneye *et al* [4] (*p*-value <0.05) was also consistent with this study. On the contrary, the 0.05 ± 0.02 HI value for 3DCRT reported by Crowe *et al* [16] was lower than the HI value for IMRT value of 0.08 ± 0.05, which shows that the 3DCRT technique had better homogeneity. A similar study by Al-Shareef *et al* [17] reported better homogeneity in 3DCRT (mean HI: 0.095 ± 0.01) than IMRT (mean HI: 0.14 ± 0.02).

### Conformity index

The study statistically compared the CI for the two techniques using the ICRU 83 formulae. In general, CI values closer to 1 indicate better conformity and this invariably translates into decreased dose to OAR without compromising doses to the target volume. This study reported a lower mean CI of 1.257 ± 0.112 for IMRT compared to 1.302 ± 0.196 for 3DCRT. Hence, the IMRT technique was more conformal than the 3DCRT approach. This was statistically significant. Statistically, the paired *t-*test analysis established a significant association between the two techniques (*p* = 0.037). The CI for this study was farther away from one compared to the studies by Al-Shareef *et al* [17] (0.93 ± 0.043 and 0.97 ± 0.02 for 3DCRT and IMRT, respectively) and Adeneye *et al* [4] (0.91 ± 0.39 and 0.99 ± 0 for 3DCRT and IMRT, respectively), but nevertheless consistent with the study. The study was also consistent with the RTOG conformity reported by Crowe *et al* [16]. The results of this study are however, inconsistent with the findings by Kinhikar *et al* [18], who reported CI values of 0.97 ± 0.02 and 0.98 ± 0.02 for IMRT and 3DCRT, respectively.

### Organs at risk

The OAR in this study were the rectum, bladder and femoral heads. The D50 and D30 constraints were used to assess the bladder and rectum whereas D5 was used to assess the femoral heads as indicated in Table 2.

#### I. Rectum

For this study, the dose comparison for the rectum assessed at 50% and 30% volume showed better sparing for IMRT than for 3DCRT. The dose reduction was 10.4% and 0.35% at 50% volume and 30% for IMRT, respectively. However, the organ sparring was only statistically significant for 50% of the volume. This finding is consistent with studies by Adeneye *et al* [4]; Kinhikar *et al* [18]; Crowe *et al* [16]; and Uysal *et al* [6], who assessed different volumes of the rectum.

#### II. Bladder

This study recorded a 0.16% dose reduction for 50% volume of the bladder for IMRT relative to 3DCRT. However, a 30% volume of bladder for 3DCRT relative to IMRT recorded an 8% dose reduction. The paired *t*-test yielded a significant *p*-value for the 30% volume of the bladder as opposed to the 50% volume of the bladder. The findings of this study contradict those of Adeneye *et al* [4]; Kinhikar *et al* [18]; Catton *et al* [19]; Crowe *et al* [16]; and Uysal *et al* [6], who had better organ sparing for IMRT compared to 3DCRT in assessing different bladder volumes.

#### III. Femoral heads

The femoral heads were assessed using 5% volume in this study. The right and left femoral heads recorded a 12.8% and 12% dose reduction for IMRT relative to 3DCRT. These differences were significant (*p* = 0.00). This is corroborated by multiple studies that have also reported similar findings [4, 6, 16].

IMRT is a highly precise form of radiation therapy that delivers targeted radiation doses to cancerous tumours while minimising damage to surrounding healthy tissue. This study confirms adherence to standard tolerance levels for OAR regarding prostate RT at one of the largest RT centers in sub-Saharan Africa. The safety of IMRT for OARs demonstrated in this study can be guaranteed in real life patient treatment by implementing robust quality assurance programs. Comprehensive quality assurance protocols must be put in place to verify the accuracy of IMRT TP and delivery processes.

The implementation of IMRT requires the adequate training of medical professionals in the operation of IMRT equipment and TP processes. Medical professionals vital for the implementation of IMRT comprise radiation oncologists, medical physicists, dosimetrists and radiation therapists. The use of IMRT is associated with improved cancer care and patient outcomes. It is therefore vital to ensure the long-term sustainability of IMRT services in sub-Saharan Africa through reliable funding, workforce training and the integration of IMRT into broader cancer care initiatives. The results of this study demonstrate the successful utilisation of IMRT in the definitive treatment of patients with PCa in sub-Saharan Africa.

## Limitations

This study did not account for interobserver variability since the delineation of target volumes and OAR was done by multiple radiation oncologists. The study was also retrospective in nature and relied on previously created contours that had already been used in the treatment of the respective patients. Before patients’ treatment, all contours and treatment plans undergo quality assurance in a forum attended by consultant radiation oncologists, medical physicists, radiotherapists and other allied health staff. However, in this study, there was no independent audit or verification of the accuracy of the contours created by the clinicians.

## Conclusion

The results of this study demonstrate that IMRT is better than 3DCRT in the treatment of patients with PCa even in low-resource settings. The superiority of IMRT over the 3DCRT technique in PCa is evident in terms of target HI, CI and dose to OAR. Overall, this study demonstrates that IMRT may offer an enhanced therapeutic profile, potentially reducing toxicity to the patient and ensuring more precise dose delivery to the target volume compared to 3DCRT.

## Conflicts of interest

The authors declare no competing interest.

## Funding

This study did not receive any specific funding support from funding agencies in the public, commercial, or not-for-profit sectors.

## Data availability

The data used to support the findings of this study are available from the corresponding author upon reasonable request.

## Figures and Tables

**Table 1.** Parameters from the DVH for both 3DCRT and IMRT plans.

DVH parameters	IMRT	3DCRT
	Mean ± SD	Mean ± SD
D2	79.16 ± 1.72 Gy	80.10 ± 1.35 Gy
D50	77.89 ± 1.47 Gy	77.93 ± 1.00 Gy
D95	98.55 ± 1.09 Gy	96.09 ± 0.90 Gy
D98	75.97 ± 1.66 Gy	73.12 ± 1.51 Gy
TV	222.30 ± 82.19 cc	222.34 ± 82.15 cc
V95	275.96 ± 95.36 cc	284.66 ± 99.71cc
HI	0.04 ± 0.02	0.09 ± 0.02
CI	1.26 ± 0.11	1.30 ± 0.20

SD = standard deviation; D2= dose at 2%; D50 = dose at 50% volume; D95 = dose at 95% volume; D98 = dose at 98% volume; TV = target volume; V95 = volume receiving 95% of dose; HI = homogeneity index; CI = conformity index

**Table 2.** Mean radiation doses delivered to the OAR.

OAR	DVH parameters	IMRT	3DCRT
		Mean ± SD	Mean ± SD
Rectum	D50	45.78 ± 5.26	51.12±5.35
	D30	59.51 ± 6.16	59.72 ± 6.95
Bladder	D50	38.12 ± 13.35	38.18 ± 18.77
	D30	55.87 ± 13.14	51.73 ± 16.28
Right femoral head	D5	34.61 ± 6.41	39.71 ± 5.34
Left femoral head	D5	34.67 ± 6.13	39.39 ± 5.44

DVH = dose-volume histogram, IMRT = intensity-modulated radiotherapy, 3DCRT = 3-dimensional conformal radiotherapy, SD = standard deviation; D50= dose received by 50% volume; D30 = dose received by 30% volume; D5 = dose received by 5% volume.

## Appendix I

**Table A1.** Summary of the beam parameters of the various treatment plans for both 3DCRT and IMRT.

Treatment machine	Beam energy	Technique	Number of fields	Gantry angles	Beam modifier
Linac	6 MV	IMRT	7	0^0^, 50^0^, 100^0^, 150^0^, 200^0^, 250^0^ and 300^0^	MLC
		3DCRT	5	0^0^, 90^0^, 142^0^, 215^0^ and 270^0^	MLC, wedge
			4	0^0^, 90^0^, 180^0^ and 270^0^	MLC

IMRT = intensity-modulated radiotherapy, 3DCRT = 3-dimensional conformal radiotherapy, MLC – multileaf collimator, MV = megavoltage

## Appendix II

**Table A2.** The dose constraints adopted for OAR.

Protocol	Structure	3DCRT Constraints
RTOG	Rectum	D50 ≤ 71 Gy, D30 ≤ 53 Gy
	Bladder	D50 ≤ 71 Gy, D30 ≤ 53 Gy
	Femoral heads	D5 ≤ 50 Gy
RTOG – Radiation Therapy Oncology Group		
